# The biological role and molecular mechanism of transfer RNA-derived small RNAs in tumor metastasis

**DOI:** 10.3389/fonc.2025.1560943

**Published:** 2025-04-08

**Authors:** Haotian Dong, Chengyuan Ye, Xiaohan Ye, Jianing Yan, Guoliang Ye, Yongfu Shao

**Affiliations:** ^1^ Department of Gastroenterology, the First Affiliated Hospital of Ningbo University, Ningbo, China; ^2^ Health Science Center, Ningbo University, Ningbo, China

**Keywords:** tsRNA, tumor metastasis, signaling pathway, epithelial-mesenchymal transition, tumor microenvironment

## Abstract

Tumor metastasis is a significant contributor to increased cancer mortality. Transfer RNA-derived small RNAs (tsRNAs), a class of endogenous non-coding RNA molecules, play crucial functional roles in various physiological processes, including the regulation of transcription and reverse transcription, the modulation of translation processes, the modification of epigenetic inheritance, the regulation of the cell cycle, etc. Dysregulated tsRNAs are closely related to the occurrence and progression of human malignancies. Accumulating evidence indicates that the abnormal expression of tsRNAs is associated with tumor metastasis through a variety of mechanisms. Hence, we summarize the fundamental structure and biological functions of tsRNAs, with a focus on how tsRNAs influence the tumor metastasis process through downstream targets or the regulation of interactions between upstream and downstream molecules, thereby providing a novel perspective for targeted therapy for tumor metastasis.

## 1 Introduction

Tumor metastasis occurs as a result of a series of biological events that arise from interactions between tumor cells and nontumor cells. This process is commonly referred to as the invasion–metastasis cascade (1). After primary tumor cells grow and proliferate to a certain extent, they detach from the primary site and modulate the activity of adhesion factors and matrix metalloproteinases (MMPs) to penetrate the extracellular matrix (ECM) and basement membrane, subsequently entering the vasculature. When highly aggressive tumor cells reach specific secondary organs, they adhere to the endothelial cortex within blood vessels and proliferate and grow under the influence of various growth factors, ultimately leading to metastatic formation. Additionally, the tumor vascular system can provide oxygen and nutrients to metastatic tumor foci (2).

Emerging research has revealed the critical involvement of non-coding RNAs (ncRNAs) in regulating tumor metastasis, with tsRNAs recently identified as novel multifunctional regulators in this process. tsRNAs are generated through the cleavage of transfer RNAs (tRNAs) by specific nucleases. tRNAs can be categorized into two types, tRNA fragments (tRFs) and tRNA-derived stress-induced small RNAs (tiRNAs), depending on the position at which cleavage occurs (3). tsRNAs are not merely degradation products of tRNAs; they play significant roles in various biological processes, including the regulation of transcription and reverse transcription, the modulation of translation processes, and participation in epigenetic modifications, among others (4). The role of tsRNAs in tumor metastasis has emerged as a significant research focus in recent years. Mechanistically, tsRNAs influence numerous processes by interfacing with epigenetic modifiers, RNA-binding proteins, and signaling cascade proteins involved in tumor metastasis. This is achieved through the regulation of downstream target genes or the modulation of interactions between upstream and downstream molecules (5–8). Advances in high-throughput sequencing technologies have enabled systematic identification of tsRNAs, with individual members exhibiting marked heterogeneity in both molecular characteristics and biological functions. These molecules may serve as novel biomarkers for the prediction of tumor metastasis.

A large number of studies have found that abnormal expression of tsRNA can regulate the process of tumor metastasis through multiple pathways, such as gene expression regulation, epigenetic modification, metabolic regulation, tumor angiogenesis, tumor immune escape, epithelial–mesenchymal transition (EMT), and the tumor microenvironment. This review discusses the various mechanisms through which tsRNAs influence tumor metastasis at the epigenetic, transcription and posttranscription levels. We offer new insights into the molecular mechanisms underlying tumor metastasis and the role of tsRNAs in tumor diagnosis, prognosis, and targeted therapies, and fresh perspectives on research on treatment options.

## 2 Origin and classification of tsRNAs

### 2.1 Origin of tsRNAs

As ncRNAs involved in protein synthesis, tRNAs facilitate the translation of mRNA nucleotide sequences by mediating the interaction between codons and anticodons during polypeptide chain synthesis. Once the corresponding amino acid sequence is released into the ribosome, tRNAs fulfills its role. In eukaryotes, tRNAs interact with nucleases (such as Dicer) to generate tsRNAs with distinct biological functions during specific maturation processes (3). Under different stress conditions, tsRNAs with diverse functionalities can be generated through cleavage and rearrangement. Under hypoxic stimulation, the expression of tDR-0009 and tDR-7336 in breast cancer cells significantly increases, and regulating downstream signaling pathways can increase the resistance of triple-negative breast cancer to doxorubicin (9). tRNAs cleavage through the induction of angiopoietin (ANG) or knockdown of ribonuclease inhibitor 1 (RNH1) increases under arsenite-induced oxidative stress, resulting in an overall increase in tsRNA expression levels (10). Under the protection of DNA methyltransferase (Dnmt2), ANG has difficulty cleaving tRNA. Heat shock stress inactivates Dnmt2, causing tRNA to lack methylation protection and thus increasing cleavage (11). With increasing numbers of related studies, researchers have discovered more types of tsRNAs. To name them uniformly, tsRNAs are usually named according to their biological origin and breakage site. Therefore, tsRNAs can be divided into two major categories: tRNA-derived stress-induced small RNAs (tiRNAs) and tRNA-derived fragments (tRFs).

### 2.2 Classification of tsRNAs

**Figure 1:**
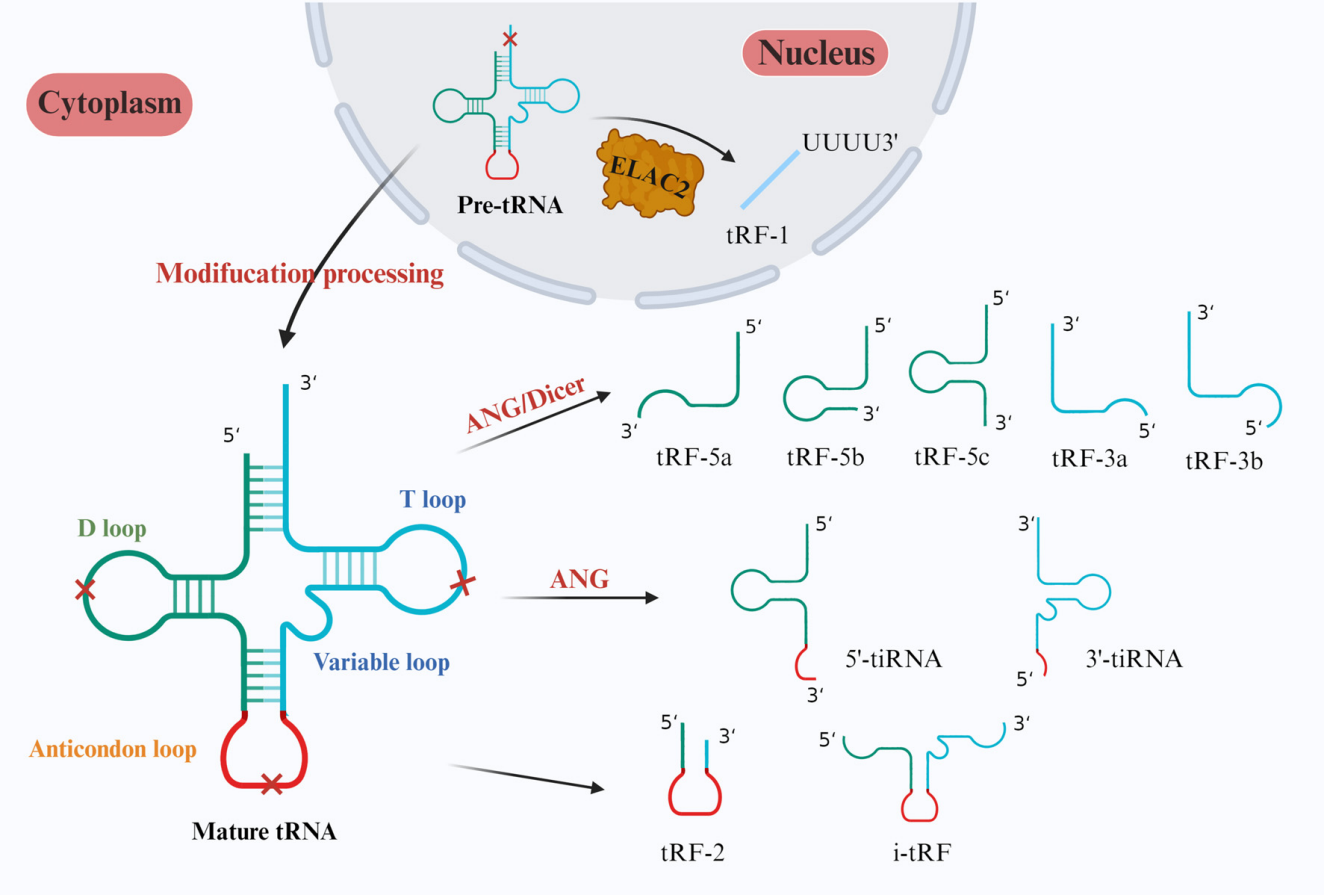
Classification of tsRNAs. In the nucleus, ELAC2 specifically cleaves pretRNAs to form tRF-1s. In mature tRNAs in the cytoplasm, Dicer cleaves the D loop to form tRF-5a, tRF-5b, and tRF-5c. ANG and Dicer then cleave the T loop to form tRF-3a and tRF-3b. Finally, tiRNAs are cleaved by ANG to form anticodon loops, which can be categorized into 5’-tiRNAs and 3’-tiRNAs. Both 2-tRFs and i-tRFs are derived from two cleavage sites in tsRNAs and typically contain anticodon loops.

tRFs generally originate from the ends of precursor or mature tRNAs and can be divided into tRF-1s, tRF-2s, tRF-3s, tRF-5s and i-tRFs according to different cutting sites (**Figure 1**). tRF-1s are derived from the 3’ end of pretRNA produced after ELAC2 cleavage and contain an RNA polymerase III transcription termination sequence (12). tRF-2s are produced by cleaving the anticodon loop under hypoxic stress; they contain only the anticodon stem and loop and do not have a conventional 5’ end or 3’ end (13). tRF-3s start from the 3’ end of tRNA and are cleaved by ANG and Dicer at the TΨC loop; they are approximately 18–22 bases in length and can be subdivided into tRF-3a and tRF-3b (13, 14). tRF-5s are derived from tRNAs starting from the 5’ end and cut by the Dicer enzyme in the D loop or the area between the D loop and the anticodon loop; they have an approximate length of 14–30 bases and can be categorized into tRF-5a, tRF-5b, and tRF-5c, depending on the cleavage site (14). i-tRFs originate from any fragment within tRNA, excluding the 5’ and 3’ ends. The naming of i-tRFs differ according to the position of the 5’ end (15).

tiRNAs are produced by cutting the anticodon loop in mature tRNA. They can be divided into 5’-tiRNAs and 3’-tiRNAs according to whether the 5’ end or 3’ end contains an anticodon loop structure. 5’-tiRNAs are derived from the 5’ end of mature tRNA to the end of the anticodon loop, and 3’-tiRNAs are derived from the anticodon loop to the 3’ end of tRNA (16). The cleavage process usually occurs under stressful conditions such as hypoxia, heat shock, oxidative stress, amino acid deficiency, viral infection, and radiation, but tiRNAs can also be generated under specific nonstressful conditions (17). tRNA modifications exert regulatory control over the biogenesis of tiRNAs (18). Besides, and the stress-induced nuclease Rny1 cleaves tRNAs in yeast to produce tiRNAs (19). In summary, the production of tiRNAs is influenced by various factors, such as the type of stress, tRNA modification, and ANG activity (15).

## 3 Biological function of tsRNAs

### 3.1 Regulation of transcription and reverse transcription

**Figure 2:**
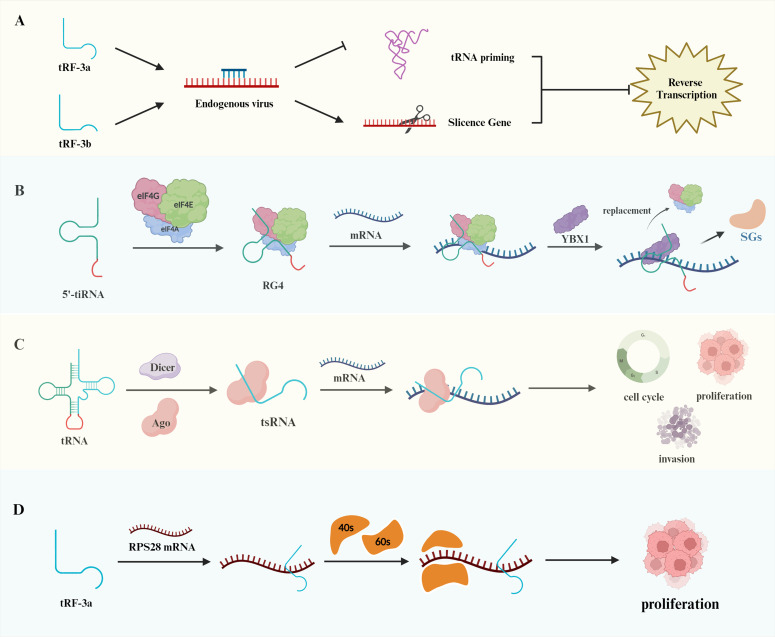
Biological functions of tRNAs. **(A)** The distinct effects of tRF-3a and tRF-3b on endogenous viruses. tRF-3a exerts its action through tRNA priming, whereas tRF-3b inhibits gene expression through silencing. Both of these mechanisms impede reverse transcription transactivation. **(B)** Binding of the terminal oligoguanine (TOG) motif at the end of the 5’-tiRNA to eIF, forming an RNA G tetramer that inhibits mRNA translation. Furthermore, YBX1 can displace eIF to further repress translation and release stress granules (SGs). **(C)** Illustration of the regulation of mRNA structure by tsRNAs, which affects the cell cycle, cell proliferation, and apoptosis. **(D)** Regulation of ribosomal subunit composition, including 40s and 60s, by tRF-3a binding to RPS28 mRNA, contributing to tumor cell proliferation.

tsRNAs play distinct roles in posttranscriptional regulation and viral reverse transcription. Dicer and the GW182 protein in the RISC complex can have the same effect when bound to tRF-3s (20). Studies in viruses have demonstrated that tRFs may have stem cell functions. In a study of respiratory syncytial virus (RSV), Deng et al. reported that tRF5-GluCTC promoted RSV proliferation by decreasing the expression of apolipoprotein E receptor-2 (ApoER2) (21). Ruggero et al. discovered a tRNA fragment detectable in leukemia virus particles, tRF-3019, which acts as a primer for human T cell lymphotropic virus type 1 (HTLV-1), enabling its reverse transcription (22). Schorn et al. reported that tRF expression was increased in mouse stem cells; the 18 nt 3’CCA tRF inhibited endogenous viral retrotransposition by blocking the reverse transcription process, whereas Dicer-dependent 22 nt tRF-3b inhibited retrotransposition by silencing the retrotransposon gene RPA1 (**Figure 2A**) (23). In early embryos and germ cells, spermatozoa have high levels of tRFs, where tRNA-GlyGCC controls the Cajal vesicle to regulate the level of histone production to ensure the complete repression of retrotransposable elements in the genome, affecting embryo growth and development processes (24).

### 3.2 Regulating the translation process

tsRNAs can regulate the translation process of proteins in a unique way. During translation initiation, tsRNAs act by affecting translation initiation factors or translation initiation complexes. Under stress conditions, 5’-tiRNAs produced by ANG cleavage of tRNAs can induce the phosphorylation of the translation initiation factor eIF2 to stall translation (25). A subsequent study revealed that eIF4G is an important scaffolding protein of the translation initiation complex and that the terminal oligoguanine (TOG) motif at the end of tsRNAs can promote the formation of the RNA G tetrameric structure; thus, this combination can effectively inhibit the translation of mRNAs and affect protein synthesis. That study also revealed that the combination of YBX1 and 5’-tiRNA displaces eIF4G in the m7G cap of mRNA and prevents translation initiation more effectively (**Figure 2B**) (26).

In addition to affecting the process of translation initiation, tsRNAs can affect the process of translation elongation by regulating the secondary structure of mRNAs. This process can be divided into AGO-dependent and AGO-independent regulation. In the AGO-dependent regulation of translational repression, short-stranded tsRNAs bind to AGO1 or AGO2, and long-stranded tsRNAs bind to AGO3 (**Figure 2C**) (27). Antisense matching of 7-mer motifs in tsRNAs to conserved sites on mRNAs reduces mRNA translation activity (28). Such as, tRF-3s and tRF-5s repress the expression of genes such as miRNAs through interactions with AGO1, AGO3 and AGO4 (29). 3′tsRNA-LeuCAG does not bind to any AGO proteins and opens the secondary structure of mRNA by binding to the corresponding site of the ribosomal protein RPS28 mRNA, which promotes the process of ribosome formation and protein translation of the mRNA. When the expression of 3′tsRNA-LeuCAG is inhibited, the reduction in RPS28 protein synthesis leads to a decrease in the number of 40S and 60S small subunits and impaired ribosome assembly, resulting in the apoptosis of cancer cells such as HeLa cells and HCT-116 cells (**Figure 2D**) (30). Furthermore, Ying et al. discovered that tRF-Gln-CTG-026 diminishes the binding of pre-40s ribosomes to TSR1, thereby inhibiting protein formation and mitigating liver damage. These findings offer a novel approach for the treatment of liver injury (31).

### 3.3 Regulation of epigenetic processes

Epigenetics refers to gene expression that affects gene transcription and translation but does not alter the DNA sequence. Studies have shown that tsRNAs can affect various epigenetic processes to regulate gene expression. PIWI proteins in germ cells can bind to piRNAs to participate in the epigenetic regulation of gene expression (32). tsRNAs are similar in length to piRNAs, and in Tetrahymena thermophila, tsRNAs are bound to the PIWI protein Twi12 to control intranuclear RNA processing (33). Zhang and colleagues reported that human monocytes differentiate into dendritic cells in response to IL-4 stimulation and that 5′tsRNA-Glu interacts with PIWIL-4 to promote histone H3K9 methylation and repress CD1A transcription (34). Transposons are DNA sequences that can potentially damage the genome; however, their activity can be effectively inhibited by epigenetic processes, such as DNA methylation or histone modification (35). 3’CCA tRF is highly expressed in Setdb1-deficient stem cells, and Setdb1 has been shown to mediate H3K9 methylation silencing of retrotransposons (24). Therefore, tsRNAs may act as epigenetic regulators of retrotransposon expression.

### 3.4 Influence on the cell cycle

The uncontrolled proliferation and apoptosis of cells represent key initiating events in tumor development. Specific fragments of tsRNAs affect the proliferation and apoptosis of cancer cells, which ultimately affects the prognosis of patients with cancer. In ER(+) breast cancer and AR(+) prostate cancer, the expression of SHOT-RNA was shown to be markedly elevated; conversely, the knockdown of SHOT-RNA expression by siRNA significantly reduced the proliferation of cancer cells (36). These findings suggest that SHOT-RNAs can stimulate the corresponding hormone receptors to promote the proliferation of breast and prostate cancer cells. In a study by Fang et al., tsRNA-5001a was markedly elevated in lung adenocarcinoma; furthermore, the overexpression of tsRNA-5001a was shown to suppress the expression of growth arrest and DNA damage-inducible 45 (GADD45), thereby facilitating the proliferation of lung cancer cells (37).

In the process of tumor development, apoptosis also plays a crucial role. The expression of GADD45, a tumor suppressor in prostate cancer, is inhibited by tRF-315, which regulates the cell cycle and prevents apoptosis in prostate cancer cells (38). Similarly, Tao et al. identified 5’tiRNA-His-GTG as a highly expressed molecule in colorectal tumors and demonstrated that reducing 5’tiRNA-His-GTG expression induces apoptosis in cancer cells; through bioinformatics analysis and rescue experiments, they revealed that 5’tiRNA-His-GTG targets the LATS2 protein, thereby upregulating the expression of antiapoptotic genes (39). Furthermore, the aberrant expression of frizzled 3 receptor (FZD3) is typically associated with the development and metastasis of malignant tumors (40). The targeting of the FZD3 protein in breast cancer cells by 5’-tiRNA-Val inhibits the Wnt/β-catenin signaling pathway, which in turn promotes cancer cell apoptosis and slows cancer progression (41).

## 4 Mechanisms by which tsRNAs affect various aspects of tumor metastasis

**Figure 3:**
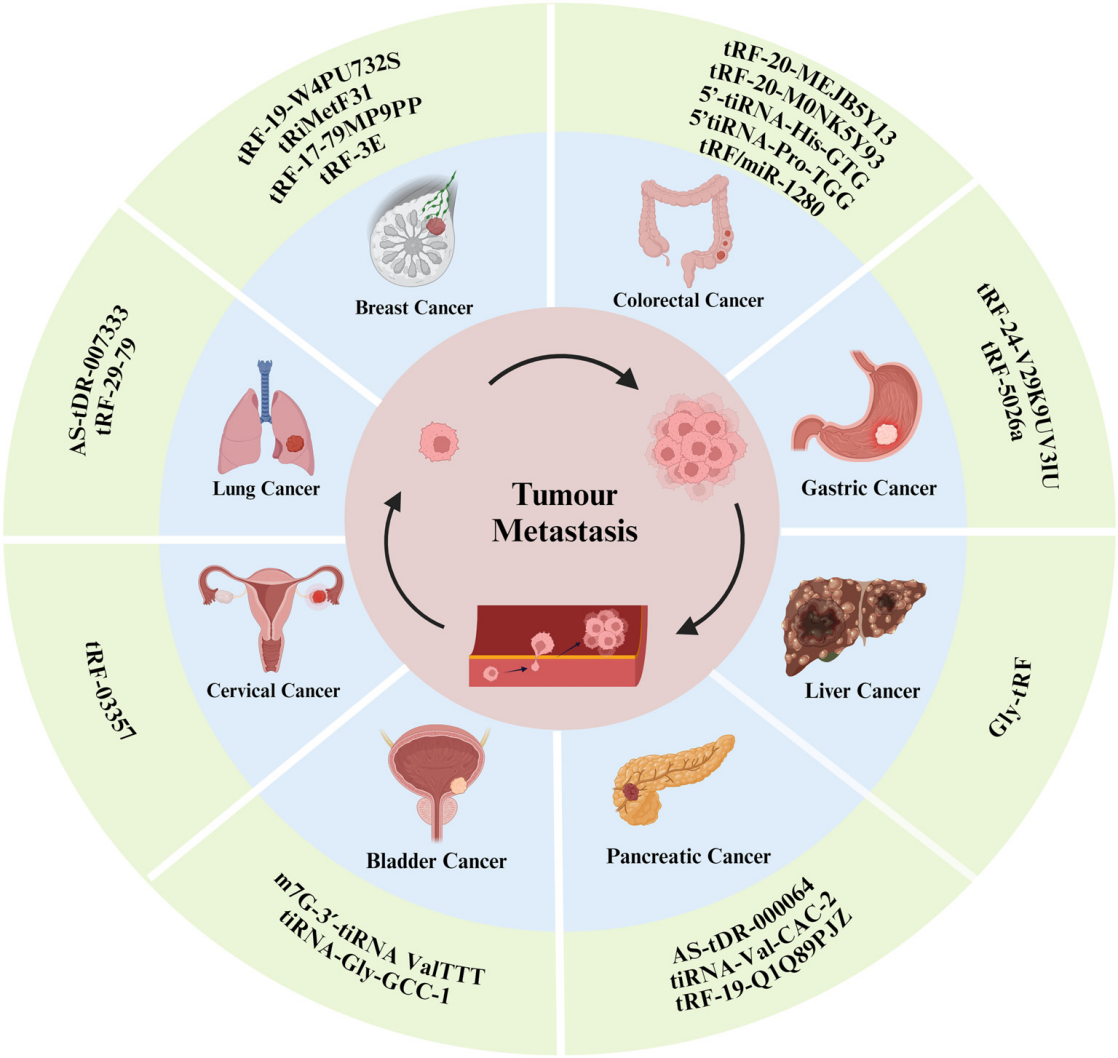
tsRNAs have been shown to influence the progression of certain types of tumor metastasis.

Tumor metastasis is a complex process involving multiple steps and factors, including cancer cell growth and proliferation, tumor angiogenesis, the epithelial–mesenchymal transition (EMT) process, immune escape, and the formation of the tumor microenvironment. tsRNAs can affect various tumor metastasis processes through different pathways (**Figure 3**). The following section outlines the molecular mechanisms of tsRNAs in tumor metastasis in detail, and we summarized this information in a **Table 1**.

**Table 1:** The effects of tsRNAs on specific tumor processes and their molecular mechanisms present in metastasis.

Molecular mechanism	Specific mechanism	tsRNA	Cancer	Function	Ref
regulating genes expression	regulating Ras expression	AS-tDR-000064	pancreatic cancer	Promotion	(43)
	activate the c-MYC	tiRNA-Val-CAC-2	pancreatic cancer	Promotion	(46)
	inhibited the translation of p53 mRNA	tRF-3E	breast cancer	Promotion	(48)
	affects PTEN	tRF-5026a	gastric cancer	Inhibition	(49)
influence epigenetic processes	histone modification	AS-tDR-007333	NSCLC	Promotion	(51)
	DNA methylation-modified	m^7^G-3′-tiRNA Val^TTT^	bladder cancer	Promotion	(52)
influence the metabolic regulation	influence glycolysis	tRF-19-Q1Q89PJZ	pancreatic cancer	Inhibition	(56)
	influence TCA cycle	tRF-30-Gly-CCC-3	thyroid cancer	Inhibition	(58)
	regulate vitamin metabolism	5’tiRNA-Pro-TGG	colorectal cancer	Inhibition	(62)
	regulate amino acid metabolism	tRF-29-79	lung adenocarcinoma	Inhibition	(64)
affect tumor angiogenesis	regulate VEGF expression	tRiMetF31	breast cancer/Glioblastoma	Inhibition	(69)
	regulate THBS1 expression	tRF-17-79MP9PP	breast cancer	Inhibition	(72)
influence tumor immune escape	regulate TLR4	tiRNA-Gly-GCC-1	bladder cancer	Promotion	(6)
	regulate NMBOX1	tRF-03357	ovarian cancer	Promotion	(77)
influence EMT	regulate Claudin-1 expression	tRF-20-M0NK5Y93	colorectal cancer	Promotion	(82)
	regulate E-calmodulin expression	tRF-19-W4PU732S	breast cancer	Promotion	(85)
		tRF-24-V29K9UV3IU	gastric cancer	Promotion	(86)
	contribute to the stem cell-like properties	Gly-tRF	hepatocellular cancer	Promotion	(90)
	loss the stem cell-like properties	tRF/miR-1280	colorectal cancer	Inhibition	(8)
affect tumor microenvironment	hypoxic environment	tRF-20-MEJB5Y13	colorectal cancer	Promotion	(93)
		tRF-20-M0NK5Y93	colorectal cancer	Promotion	(94)
		5’-tiRNA-His-GTG	colorectal cancer	Promotion	(39)
		tRF-21-RK9P4P9L0	lung adenocarcinoma	Inhibition	(96)
		tRF-19-Q1Q89PJZ	pancreatic cancer	Inhibition	(56)

### 4.1 tsRNAs affect tumor metastasis by regulating genes

At present, there are numerous comprehensive investigations into the mechanisms through which the aberrant expression of tumor-related genes influences the process of tumor invasion and metastasis. It has been demonstrated that the overexpression of oncogenes and the inactivation of antioncogenes can induce the emergence of tumor metastatic phenotypes and contribute to tumor metastasis. Certain specific tsRNAs have the capacity to regulate the expression levels of genes, thereby influencing tumor invasion and metastasis. The Ras gene has been identified as an early oncogene with a demonstrated link to tumor metastasis (42). The downstream target gene of AS-tDR-000064 is enriched in the Ras signaling pathway and has been shown to promote pancreatic cancer (PC) invasion and metastasis by regulating the expression level of the Ras gene (43). The FUBP protein exerts regulatory control over EMT during the metastasis of PC cells via the transforming growth factor beta (TGF-β)/Smad signaling pathway (44); additionally, it has been shown to activate the c-MYC upstream element (FUSE) to regulate c-MYC transcription in lung and breast cancers (45). Xiong et al. reported that the binding of tiRNA-Val-CAC-2 to FUBP1 promotes the metastasis of PC cells (46). To gain further insight into its regulatory mechanism, researchers employed a ChIP assay to determine whether FUBP1 is enriched in the c-MYC promoter region; they reported that FUBP1 accumulated at its upstream element (FUSE). To validate this finding, the researchers conducted a Transwell assay, which demonstrated that reducing FUBP1 expression restored the protein level of c-MYC and attenuated the ability of tiRNA-Val-CAC-2 to promote cancer cell metastasis. In conclusion, tiRNA-Val-CAC-2 has been shown to promote PC metastasis by inhibiting the degradation of FUBP1 and activating c-MYC transcription.

The tumor suppressor gene p53 has been shown to influence the processes of EMT and ECM formation during tumor metastasis (47). The reduced expression of tRF-3E, a tRNA-Glu-derived fragment, leads to diminished interaction with the RNA-binding protein nucleolin, which subsequently inhibits the translational process of p53 mRNA, thereby promoting the proliferation and metastasis of breast cancer cells (48). In gastric cancer, tRF-5026a directly binds to and stabilizes PTEN mRNA through complementary base pairing, resulting in upregulation of the tumor suppressor PTEN, which in turn inhibits the PI3K/Akt pathway, thereby impeding cancer cell proliferation and migration (49). In conclusion, tsRNAs can affect the process of tumor metastasis by modulating the expression of oncogenes or tumor suppressor genes.

### 4.2 tsRNAs influence epigenetic processes to regulate tumor metastasis

Epigenetic processes are intimately associated with tumor proliferation and metastasis. Recently, it has been demonstrated that tsRNAs can be employed as epigenetic regulators to influence tumor metastasis, as diagnostic targets and as potential avenues for therapeutic intervention in tumors (50). Epigenetic modifications include DNA methylation, histone modification and noncoding RNA regulation. A single epigenetic modification can influence tumor proliferation and metastasis; moreover, different epigenetic modifications can interact to enhance tumor development. Upregulated in non-small cell lung cancer tissues, AS-tDR-007333 exerts dual oncogenic mechanisms. Firstly, this tRF physically interacts with heat shock protein HSPB1 to epigenetically activate MED29 transcription through elevating histone H3K4 monomethylation (H3K4me1) and H3K27 acetylation (H3K27ac) levels at the MED29 promoter; Concurrently, it stimulates ELK4 transcription factor expression, facilitating ELK4 binding to the MED29 promoter for transcriptional reinforcement. These coordinated actions drive MED29-mediated oncogenic proliferation and metastatic dissemination of cancer cells (51).

In addition to the histone modification mechanism, Ying et al. reported a correlation between high expression of the methylation-modified tsRNA m7G-3’-tiRNA ValTTT (mtiRL) and the proliferation and metastasis of bladder cancer cells (52). Subsequent studies revealed that mtiRL promotes bladder cancer metastasis by binding to the ANXA2 protein. mtiRL enhances the binding of ANXA2 to Yes1 and facilitates the phosphorylation of ANXA2 at Tyr24, thereby stimulating the proliferation and metastasis of bladder cancer cells.

### 4.3 tsRNAs influence the metabolic regulation of tumor metastasis

**Figure 4:**
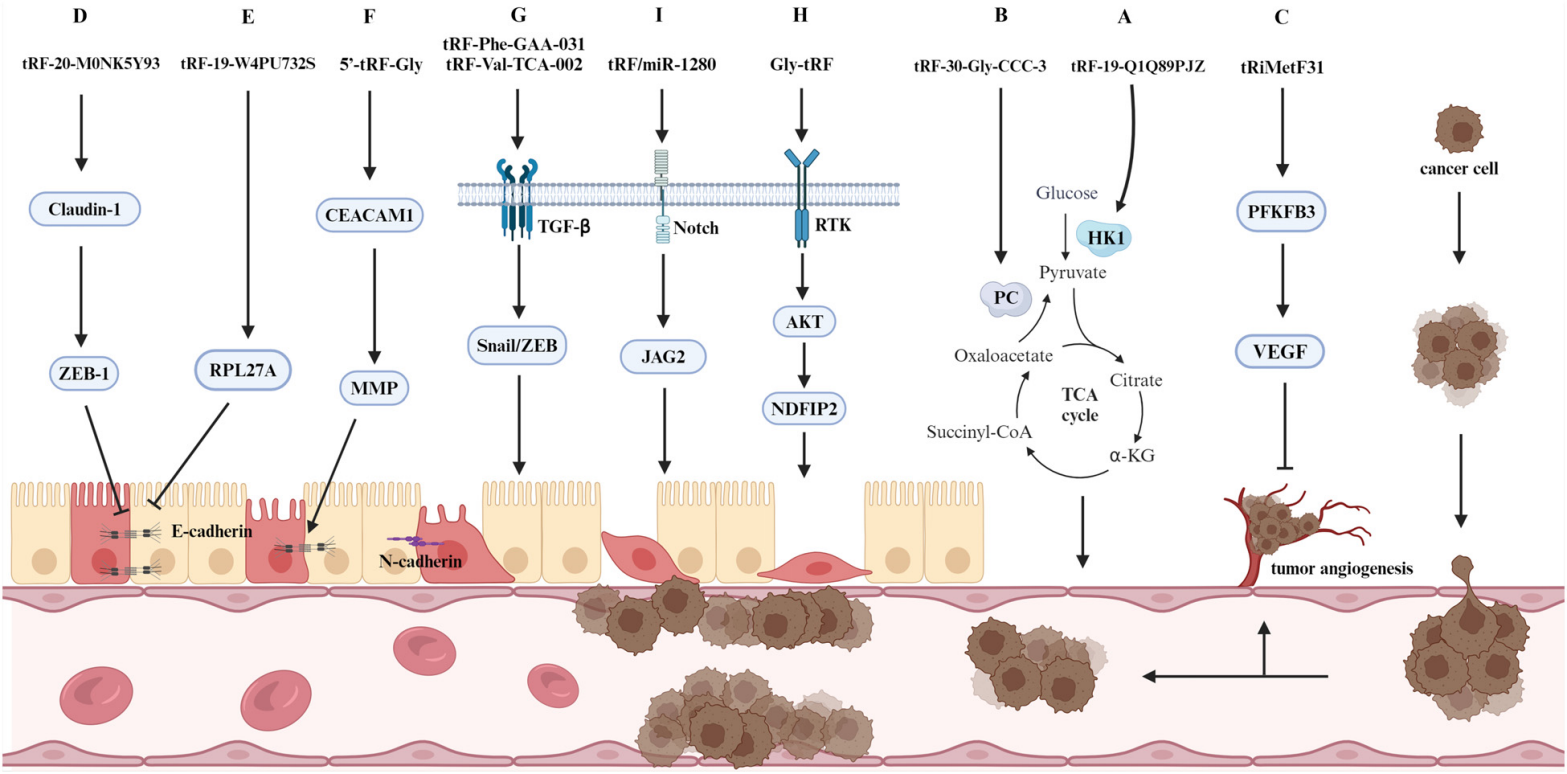
A selection of molecular mechanisms influencing tumor metastasis. The figure delineates the role of tsRNAs in tumor metastasis, specifically their involvement in regulating EMT, signaling pathways, metabolic pathways, and tumor angiogenesis.

Cancer cells develop a distinctive metabolic pattern, which differs from that of normal cells, to meet the energy demands of their aberrant proliferation (53). Glycolysis represents the most prevalent metabolic reprogramming pathway in cancer cells (54), which enables cancer cells to obtain sufficient ATP to facilitate growth, proliferation and metastasis and effectively resist apoptosis. tRF-19-Q1Q89PJZ significantly inhibits the invasive and metastatic ability of PC cells. Furthermore, the glycolysis/gluconeogenesis signaling pathway has been identified as a key factor influencing metastasis in PC cells through bioinformatics analysis. Hexokinase 1 (HK1) is a pivotal enzyme in glycolysis and is linked to prognosis in patients with PC (55). Therefore, the anti-metastatic effect of tRF-19-Q1Q89PJZ overexpression in pancreatic cancer cells is likely mediated through suppression of HK1-dependent glycolysis, which disrupts metabolic reprogramming and thereby attenuates oncogenic progression (**Figure 4A**) (56). Papillary thyroid carcinoma (PTC) is characterized by an active tricarboxylic acid (TCA) cycle (57), and tRF-30-Gly-CCC-3 is a potential oncogene in PTC that binds to the biotin-dependent enzyme pyruvate carboxylase to inhibit the production of intermediates in the TCA cycle in PTC cells and regulates the metabolic reprogramming process; this ultimately inhibits the proliferation, invasion and metastasis of PTC cells (**Figure 4B**) (58). Furthermore, it offers a novel approach to the management of PTC.

There has been a progressive expansion in the field of research investigating the correlation between metabolic pathways associated with amino acid nutrients and vitamins and the process of tumor metastasis (59–61). Wang et al. reported elevated expression of 5’tiRNA-Pro-TGG in colorectal cancer (CRC) specimens, and 5’tiRNA-Pro-TGG targeted HPSE2 to regulate the proliferation and metastasis of cancer cells; metabolic analysis indicated that HPSE2 inhibits multiple metabolic pathways, including riboflavin and retinol, which can effectively reduce CRC development and metastasis (62). CRC consistently demonstrates profound dysregulation of amino acid metabolic networks. Existing literature demonstrates that i-tRF-Glu modulates the expression of glutamine synthetase (GLUL) and related enzymes governing Glu-to-Gln conversion, mechanistically linking this tRNA-derived fragment to the pro-metastatic metabolic reprogramming characteristic of colorectal cancer progression (63). SLC1A5 is a core transporter protein for glutamine metabolism. The binding of tRF-29-79 to PTBP1 affects the stability of SLC1A5 mRNA and mediates glutamine metabolism in lung adenocarcinoma, thereby inhibiting cancer cell proliferation and metastasis (64).

The use of drugs that affect glycolytic and mitochondrial metabolic pathways in cancer cells represents an effective anticancer strategy compared with more general forms of therapy (65). Subsequent research should focus on the identification of tumor markers that influence metabolic pathways.

### 4.4 tsRNAs affect tumor angiogenesis during metastasis

In solid tumors, an increase in the number of tumor vessels is associated with an increased probability of metastasis and a poorer prognosis (66). Numerous different biomolecules have been identified as influencing tumor angiogenesis. These include growth factors, adhesion factors, proteases, angiopoietins and transcription factors, among others (67). tRiMetF31 is a miR-34a-guided tRNA-derived fragment, and its downstream target 6-phosphofructo-2-kinase/fructose-2,6-bisphosphatase 3 (PFKFB3) reduces cancer cell invasion and metastasis by normalizing the tumor vasculature (**Figure 4C**) (68). In breast cancer, tRiMetF31 has been mechanistically demonstrated to suppress tumor angiogenesis and metastatic dissemination through direct targeting of PFKFB3. This anti-angiogenic activity is primarily mediated by transcriptional downregulation of vascular endothelial growth factor (VEGF), consequently impairing endothelial cell sprouting and neovascularization (**Figure 4I**) (69). Similarly, the tRiMetF31/PFKFB3 axis has been shown to exert an inhibitory effect on glioblastoma metastasis and angiogenesis (70). THBS1 is a protein released by activated platelets and has been shown to play an inhibitory role in tumor metastasis through its angiogenic properties (71). In their study, Mo et al. demonstrated that tRF-17-79MP9PP inhibits the THBS1-mediated TGF-β1/Smad3 pathway, thereby suppressing the proliferation and metastasis of breast cancer cells (72). In CRC, ANG expression is typically linked to the metastasis of cancer cells. 5’-tiRNA-Val expression is elevated in CRC tissues and regulates tumor metastasis via the tiRNA-ANG axis (5). This finding offers a novel target for the diagnosis and treatment of metastatic CRC.

In recent years, highly selective targeted drugs have demonstrated some efficacy in antitumor angiogenesis therapy. For example, tRF-1001 activates the downstream targets RBPJ and MAML1 by silencing METLL3, which in turn inhibits vascular endothelial cells and reduces pathological angiogenesis. tRF-1001 can therefore be employed as a targeted therapeutic agent to limit ocular pathological neovascularization (73).

### 4.5 tsRNAs influence tumor immune escape during metastasis

During the process of tumor metastasis, the body’s immune system is capable of recognizing immunogenic cancer cells and limiting their growth and metastasis. However, cancer cells with high metastatic potential can evade immune system recognition and attack through the utilization of specialized molecular mechanisms. This process is referred to as tumor immune escape.

In a recent study, Qin and colleagues reported a potential correlation between the metastasis of uroepithelial bladder cancer (UBC) and tumor immune escape involving tiRNA-Gly-GCC-1 (6). Toll-like receptor 4 (TLR4) has been identified as a potential target gene for UBC and has been shown to increase B7-H1 expression in bladder cancer cells through the ERK signaling pathway, thereby promoting the metastasis of cancer cells (74). tiRNA-Gly-GCC-1 binds to its target, TLR4, and acts as a ‘sponge’, activating the immune escape of cancer cells and promoting the proliferation and metastasis of UBC. High-grade plasmacytoid ovarian cancer (HGSOC) is typically diagnosed at an advanced stage because of its insidious onset and high metastatic potential (75). In a previous study, the expression of the transcription factor HMBOX1 was shown to be markedly lower in HGSOC cells than in normal ovarian epithelial cells, and HMBOX1 was shown to impede tumor progression by curbing immune evasion (76). Zhang et al. demonstrated that tRF-03357 can facilitate the proliferative migration of ovarian cancer cells by downregulating the expression of the target gene NMBOX1 via the MAPK and Wnt signaling pathways (77), suggesting a novel approach for the early diagnosis and targeted treatment of ovarian cancer.

The tumor microenvironment is characterized by the presence of immunosuppressive immune cells that promote cancer cell proliferation and metastasis. T cells are the primary effector cells of antitumor immunity; they recognize tumor-specific antigens on the surface of cancer cells and activate cytotoxic T lymphocytes (CTLs) or T helper (Th) cells to inhibit cancer cell immune escape (78). Gao et al. identified a correlation between tRDFs in lung cancer and the tumor immune microenvironment (TIME). They reported that the increased expression of 5’-tRDF accelerated the activation of memory CD4+ T cells and memory CD8+ T cells in the TIME and that the enrichment of tRNA-Ala-TGC-3-1 in the T cell receptor signaling pathway indicates that tRDFs may play a role in tumor immunomodulation (79). These findings suggest new avenues for influencing the process of tumor metastasis and provide insights into potential immunotherapy strategies.

### 4.6 Relationships between tsRNAs and EMT during tumor metastasis

EMT represents a pivotal step in cancer cell metastasis; it not only affects alterations in adhesion molecules and related signaling pathways but also endows cancer cells with stem cell-like properties, thereby facilitating the rapid proliferation of cancer cells within metastatic foci (80). Calreticulin is primarily responsible for the transformation of micromere cells into tightly bonded cells. The different types of calreticulin and their expression levels significantly influence the EMT process (81). Luan et al. reported that the reduced expression of tRF-20-M0NK5Y93 upregulated Claudin-1 expression and thus promoted CRC metastasis (**Figure 4D**) (82). Subsequent studies have demonstrated that the transmembrane protein Claudin-1 can regulate EMT marker genes, thereby influencing the EMT process. The specific effects observed depend on the cellular environment (83). In CRC cells, Claudin-1 upregulates ZEB-1, which in turn inhibits the expression of E-calmodulin and thus promotes the EMT process (84). Furthermore, the expression of tRF-19-W4PU732S is elevated in breast cancer and is associated with the proliferation and metastatic process of cancer cells. During the metastatic process, tRF-19-W4PU732S also inhibited the activity of downstream RPL27A, thereby inhibiting the expression of E-calmodulin, which in turn inhibited apoptosis and promoted metastasis (**Figure 4E**) (85). Similarly, the knockdown of tRF-24-V29K9UV3IU in gastric cancer cells resulted in a reduction in the expression of E-calmodulin, an increase in the expression of N-calmodulin and waveform protein, and the acceleration of the EMT process in cancer cells, which in turn led to an increase in the invasiveness of gastric cancer (86). Furthermore, the expression of calmodulin is also linked to matrix metalloproteinase (MMP) activity. Liu et al. demonstrated that 5’-tRF-Gly facilitates the metastasis of hepatocellular carcinoma (HCC) cells by silencing CEACAM1 (87). The detection of EMT markers revealed that CEACAM1 affects downstream proteins, including MMP2, the cell cycle protein D1 and E-calmodulin. TGF-β, which acts as an inducer, also affects the expression of calmodulin, waveform protein and fibronectin during EMT (**Figure 4F**). The elevated expression of tRF-Phe-GAA-031 and tRF-Val-TCA-002 in CRC has been found to be associated with a poor prognosis; furthermore, this tRF has been shown to promote the EMT process in tumor metastasis by affecting TGF-β expression (**Figure 4G**) (88).

Cancer cells that have undergone EMT exhibit properties analogous to those of tumor stem cells, including the promotion of metastasis, invasion and drug resistance (89). The expression of Gly-tRF is increased in HCC and facilitates the metastasis of HCC cells (90). NDFIP2 is a direct target of Gly-tRF, and the overexpression of NDFIP2 modulates the AKT signaling pathway, thereby inhibiting the promoting effect of Gly-tRF on tumor metastasis. In particular, Gly-tRF was shown to increase the expression of stem cell-like phenotype markers, thereby contributing to the stem cell-like properties of metastatic HCC cells. Additionally, NDFIP2 was shown to influence the expression of calmodulin, as determined via EMT core marker assays (**Figure 4H**). The metastasis of CRC is typically associated with EMT. tRF/miR-1280 binds to and inhibits the expression of the downstream target gene of the Notch pathway, JAG2, which results in the loss of stemness in cancer cells and a reduction in the number of cancer cells with EMT phenotypes, thus inhibiting the growth and metastasis of CRC (**Figure 4I**) (8).

### 4.7 tsRNAs affect tumor microenvironment formation during metastasis

The tumor microenvironment is a complex system comprising the metabolic environment surrounding tissues and the cellular environment, which includes multiple stromal cells (91). The metabolic environment is characterized by tissue hypoxia and acidosis. This section focuses on the effects of tsRNAs on tumor metastasis within a hypoxic environment.

Hypoxia-inducible factor 1 alpha (HIF-1α) plays a pivotal role in regulating tissue production in hypoxic environments (92). Under hypoxic conditions, the overexpression of Dicer1 has been shown to promote the metastasis of CRC. The mechanism by which this occurs is thought to involve the induction of the expression of EMT-associated factors. Luan et al. reported that the knockdown of tRF-20-MEJB5Y13 inhibited the overexpression of Dicer1 and thus the proliferation and metastasis of CRC (93). In addition, subsequent studies revealed that tRF-20-M0NK5Y93 influences tumor metastasis not only through its impact on the EMT process but also through the metastasis of CRC cells in a hypoxic environment. In a hypoxic environment, tRFs can bind to specific sequences in long noncoding RNAs (lncRNAs), thereby negatively regulating the expression of MATLA1, inducing the SRSF2-activated selective shearing of SMC1A mRNA and promoting the metastasis of CRC (94). Similarly, in CRC, HIF-1α has been shown to accelerate ANG transcription and promote tRNA cleavage, which has been shown to increase the expression level of 5’-tiRNA-His-GTG; this, in turn, activates its downstream target, i.e., the LATS2/Hippo axis, thereby promoting the proliferation and metastasis of CRC (39). The interaction between HIF-1α and the Notch pathway has been shown to influence the process of lung tumor metastasis (95). In the tsRNA–mRNA network established by Wang et al., tRF-21-RK9P4P9L0 was found to be associated with lung adenocarcinoma (LUAD) invasion, metastasis and prognosis. Furthermore, the inhibition of tRF-21-RK9P4P9L0 expression promoted the expression of Notch1, thereby reducing the invasiveness and metastasis of lung adenocarcinoma cells. Conversely, the expression of Notch1 was shown to increase further under hypoxic conditions (96). A reduction in tRF-19-Q1Q89PJZ levels in hypoxic environments has been shown to contribute to increased HK1 transcript levels, thereby alleviating the inhibitory effect of tRF-19-Q1Q89PJZ on PC metastasis (56). These findings indicate that the HIF-1α-mediated hypoxic environment influences both the regulatory function of tsRNAs and the proliferative capacity of tumor metastasis.

In addition to investigating the effects of hypoxic environments on tumor metastasis, hypoxic conditions have also been found to be responsible for the increased resistance of tumors to chemotherapy. The metastatic tRNA-derived fragments tDR-0009 and tDR-7336, which are formed under hypoxia-specific induction, have been shown to be significantly increased in triple-negative breast cancer (TNBC) cells (9). Bioinformatics analysis revealed that STAT3 is the most frequently interacting downstream target of tDR-0009 and tDR-7336. STAT3 has been shown to activate the transcriptional function of HIF-1α, thereby conferring hypoxia-induced chemoresistance in cancer cells (97). This finding offers a novel avenue for research aimed at enhancing the efficacy of targeted therapy with chemotherapeutic agents and improving the prognosis of individuals with tumors.

## 5 Conclusions and outlook

Previous research has indicated that metastases are responsible for more than 90% of mortality from malignant tumors and cause varying degrees of damage to other organs (1). Slowing tumor metastasis and improving cancer patient prognosis have become key research areas within the contemporary medical community. tsRNAs play a role in the proliferation and metastasis of malignant tumors due to their structural stability and specificity. Specific tsRNAs associated with metastasis can be screened out by high-throughput RNA sequencing, such as the highly expressed tRF-phe-GAA-031 and tRF-VAL-TCA-002, which promote the dysregulation of EMT process and affect the metastasis of CRC (88). In addition, relevant cell function and mechanism analysis found that tsRNA is expected to be a biomarker for detecting the possibility of tumor metastasis and predicting prognosis. Nevertheless, the field of research remains constrained by several limitations.

First, there is no uniformity in the nomenclature used. The cleavage site for tRNA varies, resulting in a diverse variety of tsRNAs. The conventional nomenclature is based on the source and type of tsRNA without a comprehensive classification system, which has led to the inadvertent exclusion of numerous research-valuable tsRNAs. It is therefore necessary to establish a relatively unified naming system for tsRNAs. Second, although the tsRNA database contains a variety of tRFs and tiRNAs that meet the criteria for research, the data related to tsRNAs in specific cancers are still limited and require further updates to meet the needs of future research. Third, the structure and function of tsRNAs are determined by different chemical modifications. It is yet to be determined whether the effects of these modifications on tsRNAs are correlated with the processes of tumor proliferation and metastasis. Finally, studies on the mechanism of the role of tsRNAs in tumor metastasis are not yet fully comprehensive; for example, it is not known whether tsRNAs regulate DNA methylation to affect tumor metastasis. Moreover, most of the studies are at the theoretical level, and there is a paucity of clinical translational studies. Therefore, further research into the molecular mechanism by which tsRNAs affect tumor metastasis is warranted. The last, emerging evidence underscores the multifaceted regulatory circuits of tsRNAs in tumor metastasis, where context-dependent expression patterns dictate dichotomous biological outcomes. For instance, tRF-3E has been shown to promote breast cancer cell metastasis through NCL binding (48), whereas tRF-17-79MP9PP demonstrates pro-metastatic suppress in the same malignancy (72). Furthermore, through modulation of EMT processes in colorectal cancer metastasis, three identified tsRNAs - tRF-Phe-GAA-031, tRF-Val-TCA-002, and tRF/miR-1280 - demonstrated diametrically opposed regulatory outcomes (8, 88).

In conclusion, recent studies have shown that tsRNAs can influence tumorigenesis and metastasis through the regulation of gene expression, epigenetic processes, the tumor microenvironment, the EMT process and other mechanisms. It is anticipated that tsRNAs will emerge as biomarkers for the clinical screening, prognostic assessment and targeted therapy of tumors. With the advent of high-throughput sequencing technology, the detection of tsRNAs has increased, facilitating further investigation into their potential to modulate tumor metastasis.
